# Optimization of Curcumin Nanocrystals as Promising Strategy for Nose-to-Brain Delivery Application

**DOI:** 10.3390/pharmaceutics12050476

**Published:** 2020-05-23

**Authors:** Angela Bonaccorso, Maria Rosa Gigliobianco, Rosalia Pellitteri, Debora Santonocito, Claudia Carbone, Piera Di Martino, Giovanni Puglisi, Teresa Musumeci

**Affiliations:** 1 Department of Drug Sciences, University of Catania, V.le Andrea Doria, 6, 95125 Catania, Italy; debora.santonocito@outlook.it (D.S.); ccarbone@unict.it (C.C.); puglisig@unict.it (G.P.); tmusumec@unict.it (T.M.); 2 School of Pharmacy, University of Camerino, Via. S. Agostino 1, 62032 Camerino (MC), Italy; maria.gigliobianco@unicam.it (M.R.G.); piera.dimartino@unicam.it (P.D.M.); 3 Institute for Biomedical Research and Innovation, National Research Council, Via Paolo Gaifami 18, 95126 Catania, Italy; rosaliamariacristina.pellitteri@cnr.it

**Keywords:** natural compound, experimental design, response surface methodology, sonoprecipitation, crystallinity, amorphism, bottom-up, Box Behnken design, olfactory ensheathing cells

## Abstract

Intranasal (IN) drug delivery is recognized to be an innovative strategy to deliver drugs to the Central Nervous System. One of the main limitations of IN dosing is the low volume of drug that can be administered. Accordingly, two requirements are necessary: the drug should be active at a low dosage, and the drug solubility in water must be high enough to accommodate the required dose. Drug nanocrystals may overcome these limitations; thus, curcumin was selected as a model drug to prepare nanocrystals for potential IN administration. With this aim, we designed curcumin nanocrystals (NCs) by using Box Behnken design. A total of 51 formulations were prepared by the sonoprecipitation method. Once we assessed the influence of the independent variables on nanocrystals’ mean diameter, the formulation was optimized based on the desirability function. The optimized formulation was characterized from a physico-chemical point of view to evaluate the mean size, zeta potential, polidispersity index, pH, osmolarity, morphology, thermotropic behavior and the degree of crystallinity. Finally, the cellular uptake of curcumin and curcumin NCs was evaluated on Olfactory Ensheathing Cells (OECs). Our results showed that the OECs efficiently took up the NCs compared to the free curcumin, showing that NCs can ameliorate drug permeability.

## 1. Introduction

Intranasal (IN) drug delivery is known to be a convenient and alternative route to oral and parenteral delivery. It can be used for local, systemic and as Central Nervous System (CNS) drug delivery [1]. For instance, localized nasal drug delivery is commonly used to treat conditions related to the nasal cavity, such as rhinitis and allergies [2]. In recent years, achieving a systemic drug action using the nose as the entry portal into the body has received more attention, but a very attractive strategy is the potential use of nasal delivery to circumvent the blood–brain barrier (BBB) allowing direct drug delivery in the CNS [3]. Even if the mechanism by which drugs sprayed into the nose may reach the brain is not fully understood [4,5] different pathways seems to be involved, as detailed in recent published reviews [6,7].

Briefly, nerve pathways (olfactory and trigeminal) connecting nasal passages to the brain and spinal cord, along with the pathways involving cerebrospinal fluid and lymphatic systems, have been implicated in the delivery of molecules from nasal cavity to the CNS. A combination of these pathways is responsible, although one pathway may predominate. In particular, nose-to-brain delivery may occur via the olfactory neuroepithelium by paracellular, transcellular or neuronal transport. The paracellular pathway is slow and passive and refers to the transfer of hydrophilic molecules across an epithelium through tight junctions between sustentacular cells or the clefts between sustentacular cells and olfactory neurons [8]. Lipophilic drugs can be transported through the transcellular process across the sustentacular cells, most likely by receptor-mediated endocytosis, fluid phase endocytosis, or by passive diffusion. Finally, the neuronal pathway consists of the transport of the substances into the neuronal cell by endocytosis or pinocytosis and transported by intracellular axonal transport to the olfactory bulb. The trigeminal nerve innervates the respiratory and olfactory epithelium of the nasal cavity, enters the CNS in the pons, and a small portion of the trigeminal nerve also terminates in the olfactory bulb. This pathway plays a key role in the distribution of intranasally administered drugs to the caudal brain regions and the spinal cord. Moreover, part of the trigeminal neural pathway enters the brain through the cribriform plate alongside the olfactory pathway, thus, it is difficult to distinguish whether intranasally administered drugs reach the olfactory bulb and other rostral brain areas via the olfactory or trigeminal pathways or if both are involved [8]. In addition to these direct pathways, transport may also occur via blood vasculature, lymphatics, and cerebrospinal fluid present in the nasal mucosa tissue which is highly vascularized. However, many problems may arise with systemic delivery due to drug elimination via hepatic and renal mechanisms, and some other limiting factors such as the BBB, drug binding to plasma proteins, degradation by plasma proteases, and potential peripheral side effects [9].

Currently, multiple types of formulations are used to administer drugs via the nasal route, which include sprays, drops, powders, gels and inserts [10]. Only recently new devices have been designed to target the delivery of sprays or powders to the olfactory region of the nose, which is one of the pathways implicated in the delivery of the drug directly to the CNS [3]. In order to overcome drug limitations, in term of physico-chemical properties suitable for intranasal administration (i.e., molecular weight, solubility, lipophilicity, degree of dissociation, stability, dose, and volume of dose administered) many encapsulation technologies have been developed [11,12]. If, on the one hand, the incorporation of drugs in nanocarriers can be advantageous and resolutive in improving some of these drug properties, on the other, some limits may still persist, such as the low volume of drug nanosuspension necessary for this administration route. One of the main limitations of intranasal dosing is the low volume of drug that can be administered, with ~100 µL per nostril the optimum amount in adults. Higher volumes are prone to drip out immediately. Therefore, the drug dose should fit into a volume of roughly 100–200 µL when both nostrils are sprayed. Accordingly, two conditions are necessary: the drug should be effective at low dosage and the drug solubility in water must be high enough to accommodate an adequate dose, necessitating highly concentrated nasal drug solutions [8,13]. In order to overcome this issue, we hypothesized the use of drug nanocrystals (NCs) for nose to brain delivery, thus providing the possibility to administer the maximum dose of the drug in the lowest formulation volume using curcumin as a model drug.

Nanocrystals refer to particles of active pharmaceutical ingredients (APIs) characterized by a crystalline (including amorphous form) character with a small amount of surfactant or polymeric material in the submicron or nanoscale range, without any matrix material. NCs can reach almost 100% drug loading and can be formulated into all kinds of dosage forms such as tablet, pellet, capsule and nanosuspension. The salient advantage of NCs is the fact that they can be delivered through various administration routes, like oral, intravenous, ocular and others [14].

Curcumin was selected for its multiple advantageous effects (i.e., antioxidant, anti-inflammatory, antimicrobial, anticancer) but also for its nature, belonging to class IV of the biopharmaceutics classification system (BCS) (poorly soluble and poorly permeable) which is a prerogative for NCs preparation [15]. Typically, to overcome these drawbacks, different strategies of micro-and nano-encapsulation in lipid and polymeric matrices have been investigated [16,17].

Taking into account the limits of curcumin when administered in free form, and the difficulties of reaching the brain after intranasal administration, some authors have proposed nanotechnological formulations (i.e., nanoparticles, micelle, microemulsion) for the delivery of curcumin to the brain by this route [18,19,20]. For example, Shinde and Devarajan (2017) disclosed microemulsions of curcumin with docosahexaenoic acid-rich oil for targeted delivery to the brain. The authors found that brain concentrations following intranasal administration were substantially higher, as evidenced in the higher Cmax and AUC, and sustained compared to corresponding intravenous formulations signifying nose to brain targeting [21]. In the study of Zhuang et al. (2011), exosomes were investigated to encapsulate curcumin or a signal transducer and activator of transcription 3 (Stat3) inhibitor. Exosomes were delivered noninvasively to microglia cells via an intranasal route. The authors concluded that more fundamental studies are required to determine the exact transportation route of exosomes from the olfactory region to the brain and their subsequent clearance [22]. Chen et al. (2013) developed a curcumin intranasal thermosensitive hydrogel composed of pluronic F127 and poloxamer 188 to improve drug brain targeting efficiency. The authors found and enhanced brain-uptake efficiency [23]. Madane and Mahajan (2016) designed and produced nanostructured lipid carriers (NLCs) containing curcumin for intranasal delivery to CNS for brain cancer therapy. Biodistribution studies showed higher drug concentrations in the brain after intranasal administration of NLCs than plain drug suspension [24]. Li et al. (2019) explored the intranasal fate of curcumin (Cur)-loaded polycaprolactone nanoparticles (PCL NPs) via fluorescent bioimaging strategies. The authors found that intact PCL NPs, both PEGylated and not, cannot enter into the olfactory bulb from the nasal cavity, whereas free Cur molecules that are released from the NPs can diffuse into the olfactory bulb. Both PCL NPs and PEGylated PCL NPs carrying Cur can permeate into the mucosa and the trigeminal nerves. Starting at 2 h post-administration, both intact NPs and Cur are transported into the brainstem. NPs reaching the brainstem can further distribute to other parts of the brain such as the middle brain. The authors concluded that the trigeminal nerve pathway, instead of the olfactory nerve route, dominates the nose-to-brain delivery of intact polymeric NPs [25].

Despite the fact that NCs technology has been developed widely in the pharmaceutical field over the past 20 years [14], to the best of our knowledge, the design and application of curcumin NCs for nose-to-brain delivery represents a novelty that has not been yet explored. Recent publications have investigated the use of nanocrystals/nanosuspensions for brain targeting after intranasal administration with other drugs [26,27]. Thus, the aim of this work is focused on the design and optimization of curcumin NCs by using a mathematical and statistical approach named Box Behnken design, its technological characterization through the mean size, zeta potential, polidispersity index, pH, osmolarity, morphology, thermotropic behavior and the degree of crystallinity for potential nose-to-brain delivery application. An evaluation of cellular uptake efficiency of NCs was also performed on Olfactory Ensheathing Cells (OECs), a particular type of glial cells that accompany the unmyelinated olfactory axon of receptor neurons.

## 2. Materials and Methods

### 2.1. Materials

Curcumin from Curcuma longa L., poloxamer 188 (Pluronic F-68), polysorbate 80 (Tween^®^ 80) and mannitol were purchased from Sigma-Aldrich (Stenheim, Germany); poly(vinylpyrrolidone) (PVP) was purchased from Polichimica Srl (Milan, Italy); ethanol was purchased from J.T.Baker (Deventer, Oland). All other chemical reagents, solvents and deionized water are of analytical grade. For biological studies, we used OECs from 2-day-old rat pups (Envigo RMS s.r.l., Udine, Italy). Leibowitz L-15 medium, collagenase and trypsin, Dulbecco’s modified Eagle’s medium (DMEM), fetal bovine serum (FBS), penicillin and streptomycin, and cytosine arabinoside were purchased from Sigma-Aldrich (Stenheim, Germany).

### 2.2. Experimental Design

The scheme of curcumin NCs formulations was performed using Box Behnken design. The experimental design was created using Design-Expert software (7.0.0, Stat-Ease Inc. Minneapolis, MN, USA). A response surface quadratic model was performed for the optimization of formulation variables and to evaluate the optimum level of each variable. The three-level Box–Behnken experimental design with categorical and numeric factors was employed to optimize the size of curcumin NCs (response). The design was composed of three levels (low, medium and high) and a total of 51 runs were carried out to optimize the chosen variables, such as curcumin concentration, stabilizer concentration, solvent to antisolvent ratio and type of stabilizer (poloxamer 188, Tween^®^ 80 and PVP). For the purpose of statistical computations, the four independent variables were denoted as X1, X2, X3 and X4, respectively. The factors and levels used in the experiments are listed in Table 1.

Contour plots and three-dimensional (3D) response surface graphs were generated for a diagrammatic depiction of the values of the response. Statistical analysis of data was performed by ANOVA, provided in the software.

### 2.3. Preparation of Nanocrystals by Solvent–Antisolvent Sonoprecipitation Technique

Curcumin NCs were prepared by the combination of precipitation and ultrasonication (sonoprecipitation) using a probe sonicator (Branson sonifier 450, Marshall Scientific, Hampton, NY, USA). Curcumin powder was dissolved in a 60% ethanol/water solution of different concentrations according to the above-mentioned experimental design in Table 1. The antisolvent phase was prepared by dissolving different concentrations and types of stabilizer in water (Table 1). The antisolvent was cooled in an ice water bath (~1 °C). Then, after curcumin had been completely dissolved, it was added into the precooled (~1 °C) aqueous phase (antisolvent phase) containing a stabilizer (poloxamer 188, PVP or polisorbate 80 at a specific concentration) under intense sonication using an ultrasonic probe. The probe, immersed 10 mm in the liquid, led the wave, transferring downwards and upwards under sonication at an ultrasonic power input of 400 W and an amplitude in the range 10–100% for 10 min. The ultrasonic sound burst was set at a constant frequency. During the preparation process, the temperature was kept constant by an ice water bath. NCs were spontaneously formed and turned the solution slightly turbid. The formed dispersion was kept overnight under a fume hood to evaporate the residual ethanol.

### 2.4. Particle Size, Polydispersity and Zeta potential Analysis

Photon correlation spectroscopy (PCS; Zetasizer Nano S90; Malvern Instruments, Malvern, UK) was employed to determine the particle size, polydispersity index (PDI) and zeta potential (ZP) of curcumin NCs at a detection angle of 90°, at 25 °C with a 4 mW He–Ne laser operating at 633 nm. Each value was measured in triplicate. The results are shown as mean ± standard deviation (SD). All measurements were taken in triplicate.

### 2.5. Nanocrystals Optimization

NCs optimization was performed using the “desirability tool” provided by the Design-Expert^®^ (7.0.0, Stat-Ease Inc., Minneapolis, MN, USA) software.

The desirability function approach transforms an estimated response into a scale-free value, called desirability. The objectives of optimization can be used to maximize, minimize, or obtain the target value of the response [28]. The levels of all independent variables are thus automatically combined to identify the optimal condition within the experimental domain. Results with values ranging from zero (undesirable) to one (desirable) are obtained.

### 2.6. Lyophilization of Nanosuspension

Accurately, 0%, 5%, and 10% *w/v* mannitol as cryoprotectant were added into the nanosuspension before deep freezing. The nanosuspension, in a round-bottom flask, was frozen using a deep freezer at a temperature of −50 °C for 24 h. The samples were then freeze-dried using Edwards Modulyo freeze dryer (Thermo, Waltham, MA, USA) for 24 h at 2 mbar to produce the dry powder. The resultant lyophilized nanosuspensions were subjected to technological characterization through thermal analysis, a re-dispersibility test in water, PCS, FT–IR, and X-ray powder diffractometry (XRPD).

### 2.7. pH Evaluation

The nanosuspension pH was determined by using a pH-meter (Mettler Toledo, Columbus, OH, USA). The instrument was calibrated by using buffer solutions (pH 4.01 ± 0.02; 7.00 ± 0.02 and 10.00 ± 0.02; slope 99.8%; Mettler Toledo, Columbus, OH, USA). Prepared nanosuspensions were placed in 10 mL beakers and pH was measured at 25 °C. Measurements were performed in triplicate and results were expressed as the mean ± standard deviation.

### 2.8. Osmolarity Measurement

The osmolarity of the nanosuspensions was determined using a cryoscopic osmometer (Osmomat, mod. 030-D, Gonotec, Germany). Deionized water (consistent with the 0mOsmol point) and a 300 mOsmol/L calibration standard (consistent with the 300 mOsmol point) was used for a two-point calibration. Samples were measured in triplicate and the mean was then calculated.

### 2.9. Differential Scanning Calorimetric Analysis (DSC)

Thermal analyses were conducted for the pure curcumin, poloxamer 188, mannitol, the physical mixture (p.m) of poloxamer 188 and curcumin, and mannitol and curcumin, the p.m of curcumin, poloxamer 188, and mannitol, and freeze-dried NCs with and without mannitol using a Mettler Toledo DSC 1 STARe system equipped with a Poly-Science temperature controller (PolyScience, Niles, IL, USA). The detection system was a HSS8 high sensitivity sensor (120 gold–gold/palladium–palladium thermocouples) and the ceramic sensor (Mettler Full Range; FRS5) with 56 thermocouples. The calorimetric system was calibrated in terms of temperature and enthalpy changes by using indium and by following the procedure of the DSC 1 Mettler TA STARe instrument.

Three mg of each sample was accurately weighed and placed into aluminum crucible and sealed using an aluminum lid by a sealing machine. The thermograms were obtained at scanning rate of 5 °C/min. over a temperature range of 25–225 °C (heating) and at a scanning rate of 10 °C/min. (cooling). The thermal behaviors of the samples such as onset temperatures, melting points and endothermic and exothermic enthalpies were extrapolated using the software provided (Mettler STARe Evaluation software system (version13.00) installed on Optiplex3020 DELL).

### 2.10. Fourier-Transform Infrared (FT-IR)

FTIR spectra were recorded with a Perkin–Elmer Spectrum 100 instrument (Perkin–Elmer, Shelton, CT, USA) by total reflectance on a ZnSe crystal. The study was done in triplicate by using a scanning range from 4000 to 600 cm^−1^ with a resolution of 4 cm^−1^, averaged over twenty scans.

### 2.11. X-ray Powder Diffractometry (XRPD)

X-ray powder diffractometry (XRPD) was used to assess the solid state of the studied samples and to evaluate their physical stability. To this end, a Philips PW 1730 (Philips Electronic Instruments Corp., Mahwah, NJ, USA) was used as an X-ray generator for Cu Kα radiation (λα1 = 1.54056 Å, λα2 = 1.54430 Å). The experimental X-ray powder patterns were recorded on a Philips PH 8203. The goniometer supply was a Philips PW 1373 and the channel control was a Philips PW 1390. Data were collected in the discontinuous scan mode using a step size of 0.01° 2α. The scanned range was 2° to 40° (2θ).

### 2.12. Scanning Electron Microscopy (SEM)

Crystal morphology was determined using a Field Emission Scanning Electron Microscope (FE-SEM) (Sigma 300, Calr Zeiss Microscopy GmbH, Jena, Germany). Samples were mounted on a metal stub with double-sided adhesive tape and then sputtered in a vacuum with a chromium layer (Q150T, Quorum Technologies Ltd., Lewes, United Kingdom).

### 2.13. Re-Dispersibility in Water

Freeze-dried curcumin NCs of optimized formulation (100 mg) and pure curcumin powder (equivalent amount contained in NCs) were redispersed by placing the sample in a glass vial with deionized (DI) water by manual shaking. Very mild vortex agitation was applied to facilitate the dispersion of the freeze-dried powder when necessary. The added water was equivalent to the original volume of the aqueous nanosuspension. The formation of aggregates or precipitates was monitored visually. Particle size, PDI and ZP of the sample resuspended were recorded by PCS.

### 2.14. Drug Content

The drug content of NCs was assessed by resuspending an accurately weighed amount of the optimized freeze-dried formulation in 10 mL of ethanol. The drug content was spectrophotometrically determined at a λ max of 470 nm by using a UV–VIS 1601 spectrophotometer (Shimadzu Italia, Milan, Italy). The calibration curve for the quantitative evaluation of the curcumin was linear in the range: 51.26–1.61 μg/mL (R^2^ = 0.9997). The procedure was performed in triplicate and the average and standard deviations were calculated. The measurement of drug content was repeated at different time intervals at 0 time and after 1, 3 and 6 months of storage at room temperature.

### 2.15. Stability Study

Stability studies were performed after the storage of lyophilized NCs at room temperature for 1, 3, and 6 months. Stability was determined in terms of NCs particle size, PDI, and ZP. Moreover, the samples were analyzed for stability by DSC and drug content over the period length.

### 2.16. In Vitro Cellular Uptake Studies

OECs were isolated from 2-day old mouse pup (P2) olfactory bulbs. All the experimental procedures were carried out according to the Italian Guidelines for Animal Care (D.L. 116/92 and 26/2014), which are in compliance with the European Communities Council Directives (2010/63/EU) and were approved by the Ethical Committee at the University of Catania (Organismo Preposto al Benessere Animale, OPBA; Authorization n. 174/2017-PR). Animals were kept in a controlled environment (23 ± 1 °C, 50 ± 5% humidity) with a 12 h light–dark cycle, and food and water available ad libitum. All efforts were made to minimize animal suffering and to use the fewest number of animals possible. OECs were obtained as described by Pellitteri et al. (2007): briefly, bulbs were removed and dissected in a cold temperature (+4 °C) Leibowitz L-15 medium [29]. Then, they were digested with collagenase and trypsin. Trypsinization was stopped by adding DMEM supplemented with 10% FBS (DMEM/FBS). Cells were resuspended and plated in flasks fed with fresh, complete DMEM/FBS and an antibiotic. To reduce the number of dividing fibroblasts, cytosine arabinoside (10−5 M), an antimitotic agent, was added 24 h after the initial plating. OECs were grown on flasks and cultured in DMEM/FBS supplemented with bovine pituitary extract. Cells were incubated at 37 °C and fed twice a week. When confluence was increased, purified OECs were re-plated onto 14-mm diameter poly-*L*-lysine (PLL; 10 μg/mL) coated glass coverslips at a final density of 1 × 10^4^ cells/coverslip and fed with DMEM/FBS medium for the uptake study. Some curcumin NCs at different concentrations (0.1, 0.5 and 5.0 µM) were added in DMEM/FBS medium, while some coverslips were grown with free curcumin at the same concentration used for curcumin NCs, as the controls (CTR), all cells were incubated for 24 h at 37 °C. After incubation, OECs both with free curcumin and with NCs were fixed by exposing them to 4% paraformaldehyde in 0.1 M PBS for 30 min and images were analyzed with a Zeiss fluorescence microscope and captured with an Axiovision Imaging System.

### 2.17. Statistical Analysis

Statistical analysis was performed using Prism 6 (GraphPad Software, Inc., La Jolla, CA, USA). For the statistical analysis, we used a one-way analysis of variance (ANOVA) followed by Tukey’s multiple comparisons test for the analysis of NCs mean size and PDI before and after the freeze-drying process and for the NCs stability study. Significance was defined as *p* < 0.05.

## 3. Results and Discussion

As previously detailed, curcumin NCs were prepared by the sonoprecipitation method, a simple technique which combines precipitation and sonication. Accordingly, a preliminary investigation was performed in order to select the independent variables and the factors to keep constant during the preparation process. In fact, different parameters may affect NCs physico-chemical properties and need to be tuned [30,31]. Firstly, we defined the type, the time and the power of sonication. In particular, after a comparison between NCs prepared by using a bath sonicator and a probe sonicator, we selected the probe sonicator because it allowed us to recover smaller and more homogenous NCs (data not shown); this is possible because the probe sonicator is in direct contact with the sample, it vibrates rapidly and thereby can transfer its ultrasonic power with massive amounts of energy instead of spread energy diffusely. Then, a further screening was performed to select the amplitude (intensity) and the time of sonication and we decided to keep both values fixed at 10 min and at an output control corresponding to 80% amplitude, respectively. Once we established the constant parameters, the independent variables and their levels were introduced to build the experimental design.

Taking into consideration that NCs are drug particles surrounded by a layer of the stabilizing agent, the selection of optimum concentration of stabilizer is critical in NCs development, thus both surfactant type (categoric factor) and concentration (numeric factor) were considered as independent variables. Polymers and surfactants are used as stabilizers for pharmaceutical NCs and not only the amount, but also the type used for stabilization is very critical [32]. The physico-chemical properties of nanocrystals depend on the type of stabilizer. Not all the stabilizers are suitable for intranasal use, because their local tolerance is often unsuitable and therefore has to be carefully evaluated. We selected polysorbate 80 and poloxamer 188, together with polyvinylpyrrolidone (PVP) polymer, according to the scientific literature published in the last 10 years [33,34]. Various factors related to drug, stabilizer and dispersion medium influence the activity of the stabilizer; thus, a scientific understanding of the mechanisms involved in the stabilization of nanocrystals will help in the rational selection of stabilizers.

The second indipendent variable was the drug concentration because it has been demonstrated that this parameter can affect particle size, and optimum drug concentration is required to achieve a smaller particle size. A concentration above the optimum level leads the generation of larger particles. On the contrary, a higher drug concentration produces higher supersaturation, which results in a faster nucleation rate and thereby smaller particles. Secondly, by increasing the drug concentration, viscosity is also increased, which will hinder the diffusion between solvent and antisolvent, leading to non-uniform supersaturation, slower nucleation rates and increased particle agglomeration, and, hence, larger particles [35]. The last independent variable considered was the solvent to antisolvent ratio (S/A), since the relative amount of solvent and anti-solvent may result in a viscosity change in the system, which, in turn, can modify the characteristics of the nanocrystals; therefore, various S/A phase ratios (i.e., 1:1, 1:5, 1:10) were evaluated. The low and high levels of each of these factor have been selected according to the scientific literature [34,36].

### 3.1. Effect of Independent Variables on NC Size

The sonoprecipitation technique was adopted for the preparation of 51 formulas in a 3^4^ factorial design.

A polynomial regression analysis was performed on the response to determine the coefficients of the model terms. The predicted response of NC size is expressed by the equation in terms of actual factors.

Surfactant type poloxamer 188:
$$\begin{array}{l} \mathrm{size}=+497.27684-35.12333\times \mathrm{curc}\;\mathrm{conc}-236.19527\times \mathrm{surfact}\;\mathrm{conc}-31.73023\times \mathrm{ratio}\frac{S}{A}\\ \;-30.000\times \mathrm{curc}\;\mathrm{conc}\times \mathrm{surfact}\;\mathrm{conc}+2.51667\times \mathrm{curc}\;\mathrm{conc}\times \mathrm{ratio}\frac{S}{A}\\ \;+7.53086\times \mathrm{surfact}\;\mathrm{conc}\times \mathrm{ratio}\frac{S}{A}+6.06417\times {\mathrm{curc}\;\mathrm{conc}}^{2}\\ \;+220.27572\times {\mathrm{surfact}\;\mathrm{conc}}^{2}+1.86737\times \mathrm{ratio}\frac{S}{A^{2}} \end{array}$$
Surfactant type PVP:
$$\begin{array}{l} \mathrm{size}=+535.78754+11.58917\times \mathrm{curc}\;\mathrm{conc}-349.72305\times \mathrm{surfact}\;\mathrm{conc}-46.10800\times \mathrm{ratio}\frac{S}{A}\\ \;-30.000\times \mathrm{curc}\;\mathrm{conc}\times \mathrm{surfact}\;\mathrm{conc}+2.51667\times \mathrm{curc}\;\mathrm{conc}\times \mathrm{ratio}\frac{S}{A}\\ \;+7.53086\times \mathrm{surfact}\;\mathrm{conc}\times \mathrm{ratio}\frac{S}{A}+6.06417\times {\mathrm{curc}\;\mathrm{conc}}^{2}\\ \;+220.27572\times {\mathrm{surfact}\;\mathrm{conc}}^{2}+1.86737\times \mathrm{ratio}\frac{S}{A^{2}} \end{array}$$
Surfactant type Tween 80:
$$\begin{array}{l} \mathrm{size}=+546.05053-36.54833\times \mathrm{curc}\;\mathrm{conc}-219.30638\times \mathrm{surfact}\;\mathrm{conc}-41.79134\times \mathrm{ratio}\frac{S}{A}\\ \;-30.000\times \mathrm{curc}\;\mathrm{conc}\times \mathrm{surfact}\;\mathrm{conc}+2.51667\times \mathrm{curc}\;\mathrm{conc}\times \mathrm{ratio}\frac{S}{A}\\ \;+7.53086\times \mathrm{surfact}\;\mathrm{conc}\times \mathrm{ratio}\frac{S}{A}+6.06417\times {\mathrm{curc}\;\mathrm{conc}}^{2}\\ \;+20.27572\times {\mathrm{surfact}\;\mathrm{conc}}^{2}+1.86737\times \mathrm{ratio}\frac{S}{A^{2}} \end{array}$$

NC size was found to be in the range of 216.1 ± 13.63 to 572.8 ± 39.89 nm. The adopted model can be used for the prediction of the response in the design space. An analysis of variance (ANOVA) was further performed to discuss the suggested model and its Fisher variation ratio (F-value) and *p*-value determine if the suggested model is significant or not. A probability value (*p*-value) of less than 0.05 means the significance of the model is at the 95% confidence level. The model’s F-value of 4.76 and *p*-value less than 0.0001 indicate that the suggested model is significant.

**Figure 1:**
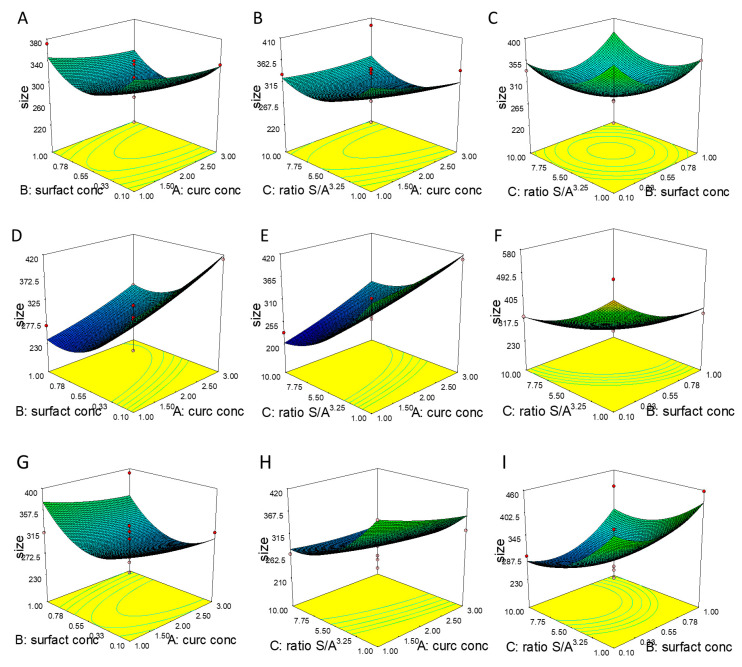
3D surface of the effect of (**A**) X1 = A: curcumin concentration (mg/mL) versus X2 = B: surfactant concentration (% *w/v*), (**B**) X1 = A: curcumin concentration (mg/mL) versus X2 = C: solvent to antisolvent (S/A) ratio (V/V) (**C**) X1 = B: surfactant concentration (% *w/v*) versus X2 = C: S/A ratio (V/V), on the nanocrystals’ (NCs) size using Poloxamer 188. 3D surface of the effect of (**D**) X1 = A: curcumin concentration (mg/mL) versus X2 = B: surfactant concentration (% *w/v*), (**E**) X1 = A: curcumin concentration (mg/mL) versus X2 = C: S/A ratio (V/V) (**F**) X1 = B: surfactant concentration (% *w/v*) versus X2 = C: S/A ratio (V/V), on the NC size using PVP. 3D surface of the effect of (**G**) X1 = A: curcumin conc. (mg/mL) versus X2 = B: surfactant concentration (% *w/v*), (**H**) X1 = A: curcumin conc. (mg/mL) versus X2 = C: S/A ratio (V/V) (**I**) X1 = B: surfactant concentration (% *w/v*) versus X2 = C: S/A ratio (V/V), on the NC size using Polysorbate 80.

**Figure 2:**
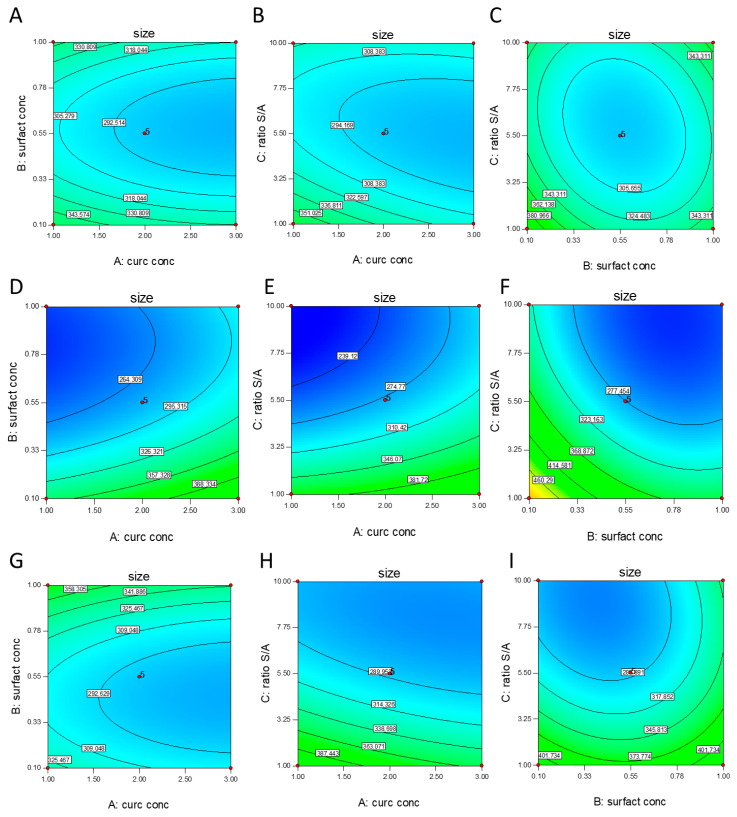
Contour plot of the effect of (**A**) X1 = A: curcumin concentration (mg/mL) versus X2 = B: surfactant concentration (% *w/v*), (**B**) X1 = A: curcumin concentration (mg/mL) versus X2 = C: S/A ratio (V/V) (**C**) X1 = B: surfactant concentration (% *w/v*) X2 = C: versus S/A ratio (V/V), on the NC size using poloxamer 188. 3D surface of the effect of (**D**) X1 = A: curcumin concentration (mg/mL) versus X2 = B: surfactant concentration (% *w/v*), (**E**) X1 = A: curcumin concentration (mg/mL) versus X2 = C: S/A ratio (V/V) (**F**) X1 = B: surfactant concentration (% *w/v*) versus X2 = C: S/A ratio (V/V), on the NC size using PVP. 3D surface of the effect of (**G**) X1 = A: curcumin conc. (mg/mL) versus X2 = B: surfactant concentration (% *w/v*), (**H**) X1 = A: curcumin conc. (mg/mL) versus X2 = C: S/A ratio (V/V) (**I**) X1 = B: surfactant concentration (% *w/v*) versus X2 = C: S/A ratio (V/V), on the NC size using Polysorbate 80.

As well as F-value and *p*-value, adequate precision (AP) and coefficient of variation (CV) can be also used as parameters for evaluating the significance of the model. AP, which is considered as the signal to noise ratio, is a measure of the range in predicted response relative to its associated error. A value greater than four is desired. Our ratio of 11.443 indicates an adequate signal. This model can be used to navigate the design space. The CV is a measure of the residual variation of the data relative to the size of the mean. It is the standard deviation divided by the dependent mean and is usually expressed as a percentage to compare the experimental precision of different experiments [37]. The value of CV for the Y_1_ model is 13.26%, which ensures a significant model according to Couto et al. (2013); if the CV is ˂10%, it is considered low and it means that the experiment presents a high precision. From 10% to 20% the CV is considered medium, implying good precision, while CV in the range between 20% and 30% is considered high, indicating low precision. Finally, if the CV is ˃30% it is considered very high, indicating very low precision [38]. As shown in Figure 1 and Figure 2, four factors at three levels were investigated in relation to NC size.

In this case B, C, BD, CD, B^2^ and C^2^ are significant model terms. The term B refers to surfactant concentration, which showed a negative coefficient (−20.11), meaning that with the increase in surfactant concentration, NC size decreases, respectively. The same trend was observed for the term C, which refers to S/A ratio (−45.72). Both B and C factors exert a significant influence on NC size, even in combination with term D, which refers to the surfactant type. The significance of quadratic terms could be signal that the relationship is non-linear. The sign merely represents the type of non linearity. A positive quadratic term (B^2^ = 44.61 and C^2^ = 37.81) could suggest that the relationship is exponential. In our study, we observed that both B and C terms are negative and the coefficients for B^2^ and C^2^ are positive indicating, that B and C have a negative effect on Y (response) until a turning point is reached, beyond which both terms showed a positive impact on Y. As detailed, the surfactant concentration exerts a strong impact on NC size, this could be explained by the decrease in surface tension by increasing the surfactant concentration, which facilitates the size reduction and stabilizes the formed NCs with inhibition of aggregation. Conversely, low amounts of surfactant are insufficient to reduce the surface free energy of new surfaces generated and their growth into bigger crystals [39]. As reported in the literature, the solvent–antisolvent ratio exerts a significant effect of the particle size of NCs suspension and, in this study, we found that particle size can be decreased by increasing the amount of antisolvent. The reason for such an observation can be associated to the supersaturation condition. Supersaturation determines crucial nanoparticle properties such as size, crystallinity, morphology and purity. Up to a critical solute concentration, a higher supersaturation leads to a decrease in particle size [40,41].

If the ratio of antisolvent to solvent is increased, the degree of supersaturation is increased as well, thus enhancing the nucleation rate and leading to the formation of small particles size. Once the nuclei are formed, particle growth occurs, which is partially hindered at higher anti-solvent volumes [35]. In fact, a high degree of supersaturation usually leads to a spatially uniform distribution of nuclei and a low crystal growth rate, which is beneficial to the formation of nanoparticles with narrow size distributions [40].

An increase in antisolvent to solvent ratio causes an increase in supersaturation through the reduction in drug solubility [42]. This is because the basic principle of solvent–antisolvent technique is that the drug is dissolved in a solvent; the solvent solution is then mixed with an antisolvent (in which the drug is insoluble). The drug precipitates as a consequence of the change of supersaturation caused by mixing the solution and the antisolvent. The key for producing small particles by antisolvent precipitation is to produce conditions that favor very rapid particle formation and little or no particle growth, such as the higher volume of antisolvent, as demonstrated by Viçosa et al. (2012) [43].

A significant impact is also highlighted for the interaction between the concentration and the type of surfactant and the S/A ratio and the type of surfactant. This result confirms that the surfactant type is a critical parameter in the formulation development, expecially for NCs, in which it represents the only excipient used. The stabilizers were selected according with the administration route. Poloxamers are often considered as “functional excipients” because they are essential components, and play an important role in the formulation [44]. They are generally considered as safe for different applications (i.e., topical, parental) and are approved by the FDA as pharmaceutical ingredients and food additives. Nowadays, their use as stabilizing agents is very common for NCs formulations [32]. In addition to its good safety profile, it has been demonstrated that poloxamer 188 can be used as an intranasal absorption enhancer of hydrophobic drugs or drugs with poor permeability [45,46]. PVP is a bulky, non-toxic, non-ionic polymer, that prevents particle aggregation via the repulsive forces that arise from its hydrophobic carbon chains that extend into solvents and interact with each other (steric hindrance effect). PVP is often a shape-control agent, promoting the growth of specific crystal faces and hindering others [47]. Moreover, PVP can be considered biocompatible and does not induce serious histological changes in the nasal mucosa; in addition, it showed a high enhancing effect on the permeation rate of lipophilic drugs, such as indomethacin from sheep nasal mucosa [48]. The third surfactant selected was Tween^®^ 80, which is a hydrophilic nonionic surfactant that has gained particular interest due to its low cost, low polarity, low toxicity and high solubilization capacity. It seems to be a promising excipient to increase drug concentration in both plasma and the brain via the intranasal route [49]. In fact, it has been demonstrated that polysorbate 80-coated nanoparticles can enhance drug distribution in the brain not only after intravenous injection, but also after oral and intranasal administration thus, polysorbate 80 might work as a message drug to increase drug distribution in the brain [49].

The significance of this model is further confirmed by Supplementary Figure S1, in which the plot of the predicted versus actual values have been displayed.

This diagnostic plot graphically shows that the predicted values are close to the experimental ones along the prediction line, showing that the model is very accurate; there is a strong correlation between the model’s predictions and its actual results [50].

### 3.2. NC Optimization

In order to optimize the process, some requirements for the factors and response need to be set, as described in Table 2. Regarding the input variables, we aimed to maximize curcumin concentration in order to obtain a final formulation with a high dose of the drug. As demonstrated by the contour plot and 3D surface (Figure 1 and Figure 2), the two main factors affecting particle size were the S/A ratio and the surfactant concentration, even though a higher S/A ratio is desired, we decided to minimize this factor in order to obtain a final formulation with a small volume. Moreover, we decided to keep surfactant concentration within the range, but, among the formulations proposed by the software, we selected the one with the highest amount of surfactant and the highest desirability.

Finally, we aimed to minimize NC size because the interaction of particles with cells is known to be strongly influenced by their size. Particles’ mean diameters affect their interactions with cells and determine the active or passive cellular internalization and intracellular localization. Mechanisms by which particles are internalized and transported into the brain are poorly defined, but in order to gain a direct transport, only particles with diameters smaller than that of olfactory axons can be intracellularly transported to the brain via the olfactory neural pathway [51]. Taking into account that the olfactory axons of humans are between 100 and 700 nm [52], we preferred to obtain NCs as small as possible in order to facilitate their transport through this direct route.

A desirability value close to one determines optimum operating conditions. The results of optimization are usually demonstrated using contour plots. Each contour shows the operating conditions needed to reach the desired response (Supplementary Figure S2).

We selected the highest value of desirability (0.872) and, at these conditions, it was predicted by Design-Expert to reach a NC size equal to 308.921. At the optimum operating conditions, the NC-optimized formulation was validated experimentally. The obtained experimental result corroborates with the predicted ones, as confirmed by the error % equal to 0.28%, which can be considered negligible [53]. Thus, the optimized NCs were produced by using Poloxmer 188 at 0.7% *w/v* and with curcumin at the highest concentration in order to obtain a concentrated formulation in a small volume suitable for intranasal administration. Once we assessed the preparation procedure, we aimed to convert the NC suspension into a dried powder due to the well-known instability of curcumin in aqueous suspension.

### 3.3. Freeze-Drying Process for NCs Long Term Storage

As is known, a major factor that limits curcumin bioavailability is its chemical instability under physiological conditions. At physiological pH, curcumin rapidly degrades to bicyclopentadione through autoxidation, with cleavage products such as bicyclopentadione, vanillin, and ferulic acid being formed [54]. Moreover, if drug particles or crystals are stored as a suspension in an aqueous medium, drug desorption, and/or drug degradation may occur. Lyophilization increases the stability and the shelf life of the finished product by preserving it in a relatively stable dry state, especially if the drug is not stable in the aqueous suspension such as curcumin [55].

**Figure 3:**
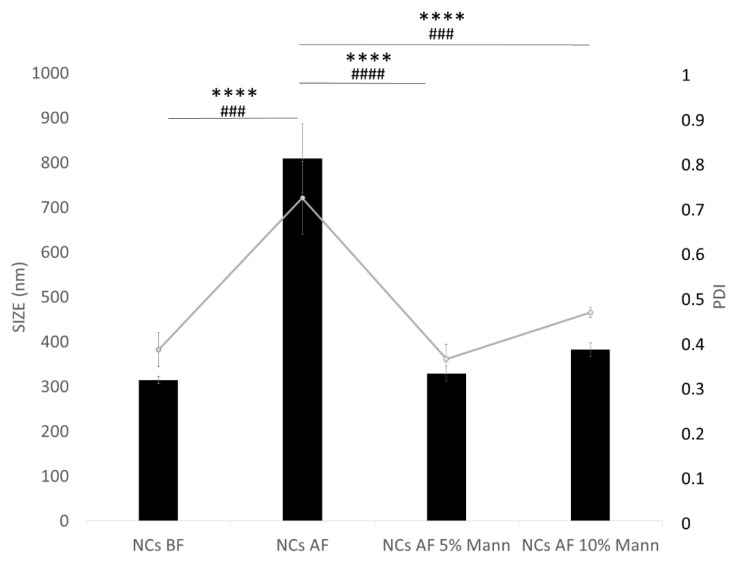
Mean size and polydispersity index (PDI) of NCs before and after freeze-drying without and with mannitol as cryoprotectant. BF = before freeze-drying; AF = after freeze-drying; 5% Mann = 5% (w/V) mannitol; 10% Mann = 10% (w/V) mannitol. Tukey’s test for NC size and PDI. The * symbol denotes statistical significance difference for size; The # symbol denotes statistical significance difference for PDI. Significance was defined as **** *p* < 0.0001.

Thus, with the aim of improving drug stability, NC nanosuspension was converted into a dry powder form by freeze-drying. After freeze drying, the easy and rapid reconstitution and unchanged particle size of the product are important features. Although the process of freeze-drying helps in establishing formulation stability, in some cases it might affect the quality of the final product [56]. As demonstrated in Figure 3 and Table 3, after lyophilization, NCs revealed an increase in particle size (Sf/Si = 2.57) and PDI values compared to the results before freeze–drying (Figure 3).

This might be due to the aggregation of particles during the freeze-drying process. In order to maintain the same particle size distribution after the freeze-drying rehydration cycle, a cryoprotectant should be added. Accordingly, mannitol was chosen as cryoprotectant agent since it showed good results with NC formulations and crystallized around the NCs, creating a protective shell to prevent aggregation [57]. Mannitol was tested at two different concentrations because the concentration of the cryoprotectant is also recognized as an important factor in the efficiency of the lyophilization process [58]. The concentration tested was selected according to the scientific literature, such as the study of Pundlikrao et al. (2013), in which 10% mannitol was sufficient to prevent fenofibrate NC agglomeration during the drying process and consequently stabilize it [57]. When the NC dispersion containing cryoprotectant is frozen below the glass transition temperature, the cryoprotectant forms a glassy/vitreous coating around the particles, protecting them against the mechanical stress of ice crystals, thereby preventing aggregation [55]. An insufficient concentration of cryoprotectant leads to the incomplete coating of the glassy matrix around particles favoring aggregation, as demonstrated in our previous study, in which different concentrations of sucrose (from 0% to 5% *w/v*) were tested to preserve poly(lactide-*co*-glycolic acid)-poly(ethylene glycol) (PLGA-PEG) nanoparticles from aggregation during the freeze-drying process [59]. In this study, mannitol at 5% and 10% *w/v* were tested and both concentrations preserved NC size, as reported by the final/initial size ratio (Sf/Si ratio) of 1.04 and 1.21 respectively, which, being less than 1.3, is considered acceptable [55,60]. Moreover, the particles size is also of great interest according to the administration route selected and, in particular, optimized NCs were homogeneous and had a suitable diameter for the IN route.

As reported in the literature, the protective effect of cryoprotectant agents is based on a surface phenomenon. Thus, the amount of cryoprotectant should be selected as a function of total superficial area correlated to the size of the particles. A critical concentration for each cryoprotector seems to exist, above which an additional amount is prejudicial in many cases [61]. Accordingly, we suppose that 5% *w/v* mannitol is sufficient to cover a NC surface exerting a cryoprotective effect.

This hypothesis is confirmed by PDI values (Figure 3), and by the presence of additional particle populations (Supplementary Figure S3C), probably due to additional components or additives (mannitol not adsorbed) in the nanosuspension (8.6%: 87.18 nm).

Therefore, according to this result, 5% *w/v* of mannitol was selected because it is enough to protect the mean diameter of the NCs, reducing at the same time the amount of excipient needed to overcome aggregation phenomenon.

Moreover, based on the literature, we can suppose that mannitol is probably one of the most commonly used bulking agents in freeze-dried pharmaceutical products because of its many positive properties with respect to crystallinity, high eutectic temperature, and matrix properties. The benefit of using mannitol is that it crystallizes during freezing and permits drying processes at higher product temperatures, and with higher sublimation rates relative to purely amorphous systems. This property promotes efficient freeze-drying and a physically stable, pharmaceutically elegant freeze-dried solid cake. Furthermore, the crystallization of mannitol during freezing could provide a scaffold on which amourphous formulation components can be dried above the critical temperature, preventing cake collapse and reducing the drying times [62].

The formulation cryoprotected with 5% mannitol showed a PDI value (0.361) strictly related to the formulation prior to the freeze-drying process (0.382). Our results can be considered acceptable, because as reported in Figure S3B, the main particles population is equal to 98%. This means that almost all particles in the nanosuspension present the same dimension and could therefore be transported through the same pathway after intranasal administration. The same consideration is true for ZP values equal to −37.4 ± 0.6 mV before lyophilization and −36.7 ± 0.4 mV after lyophilized reconstitution, demonstrating that mannitol can preserve NC homogeneity and indicating a good stability against coalescence after redispersion.

The high and negative ZP value observed is probably due to the presence of P188, which forms a sterically stabilized polymer layer. In fact, several factors affect the size and stability of NC suspensions such as stabilizer molecular weight, functional groups, and hydrophilic/hydrophobic ratio in the stabilizer molecule. The driving force for poloxamer diffusion and absorption onto the crystal surface is the hydrophobic moiety of copolymer, its attachment onto particle surfaces is based on physisorption; the high hydrophobicity of polymers facilitates fast diffusion, strong absorption and sustained time for the desorption of the polymer.

The poloxamers facilitate a decrease in ZP values with increasing molecular weight (P188 < F127) [63].

Moreover, taking into consideration our previous study, in which the influence of the surface charge of rhodamine-loaded PLGA nanoparticles (negative charge) and chitosan/PLGA nanoparticles (positive charge) was investigated on the localization in different brain sub-regions (rostral and/or caudal brain regions) during a time course after intranasal administration, we could hypothesize that the negative charge of NC could promote a potential prevalent localization in rostral brain regions [64].

Furthermore, the negative zeta potential of NCs could be beneficial for the IN route to avoid electrostatic interaction with mucin present in the nasal cavity. In fact, nanocarriers designed to target the brain via nasal administration should prevent drug loss by reducing the adherence to mucus and the residence time in the nasal cavity [51].

The redispersion of samples was evaluated after macroscopic observation, as reported in Table 3. The curcumin powder did not redisperse in water due to its high lipophilic nature, while NCs redispersed easily and after gentle shaking until a uniform dispersion with no visible macroscopic particles was obtained, as highlighted by the intensity of the orange color of the water, illustrating this phenomenon as well (data not reported).

The presence of the stabilizer together with the conversion of the drug into NC form significantly enhances the redispersibility of curcumin, demonstrating that this is a very versatile technique for this purpose [65]. Even if lyophilized NCs without cryoprotectant redisperse easily, NCs with mannitol present a more intense yellow colour and a very rapid redispersibility, probably due to the despersant activity of the soluble vitreous shell surronding NCs [66].

The optimized formulation was subjected to a physico-chemical investigation because several formulation factors should be taken into consideration to obtain the successful intranasal delivery of drugs. In addition to the concentration of the drug, the dose and volume of administration, and even the pH and osmolarity play critical roles. Formulation parameters are shown in Table 4.

No drug loss was found during the preparation method, as revealed by the drug content assay. The osmolarity and pH of the vehicle may affect local tolerance [67]. Thus, the nasal formulation should be adjusted to an appropriate pH to avoid irritation and to obtain efficient absorption. In the study of Washington et al. (2000), the authors determine the baseline physiological pH of the nasal cavity in 12 healthy volunteers and they found that the healthy human volunteers’ overall range of pH of the anterior part of the nose was 5.17 to 8.13, while that of the posterior part was 5.20 to 8.00, indicating that the average baseline human nasal pH is approximately 6.3 [43]. Thus, the stability can be achieved by the proper selection of formulation pH. However, the pH of the formulation should be near to human nasal mucosa (5.0–6.5) to prevent sneezing [68]. The NC formulation pH was found to be equal to 6.1 which is within the ideal range; in fact, pH <3 or >10 has been shown to result in tissue damage, and irritation can occur outside the physiological range. The osmolarity of the dosage form affects the nasal absorption of the drug, as was observed in the rats with a model drug. The formulation tonicity substantially affects the nasal mucosa, and thus an isotonic formulation is preferred. A formulation designed for the IN route should be isoosmotic, since hypo-osmotic formulations (<50 mOsm/kg) can improve absorption, but also enhance the potential for epithelial damage, while hyper-osmotic formulations (>900 mOsm/kg) may increase mucus secretions [3]. Freeze-dried NCs resuspended in water showed an osmolarity value of 145 mOsm/L. According to Patel et al. (2009), nasal formulations with osmolarity values within the range of 85.47–341.88 mOsmol/L can be considered acceptable because they do not harm the nasal cilia [69].

### 3.4. Physico-Chemical Characterization of Freeze Dried Optimized NCs

**Figure 4:**
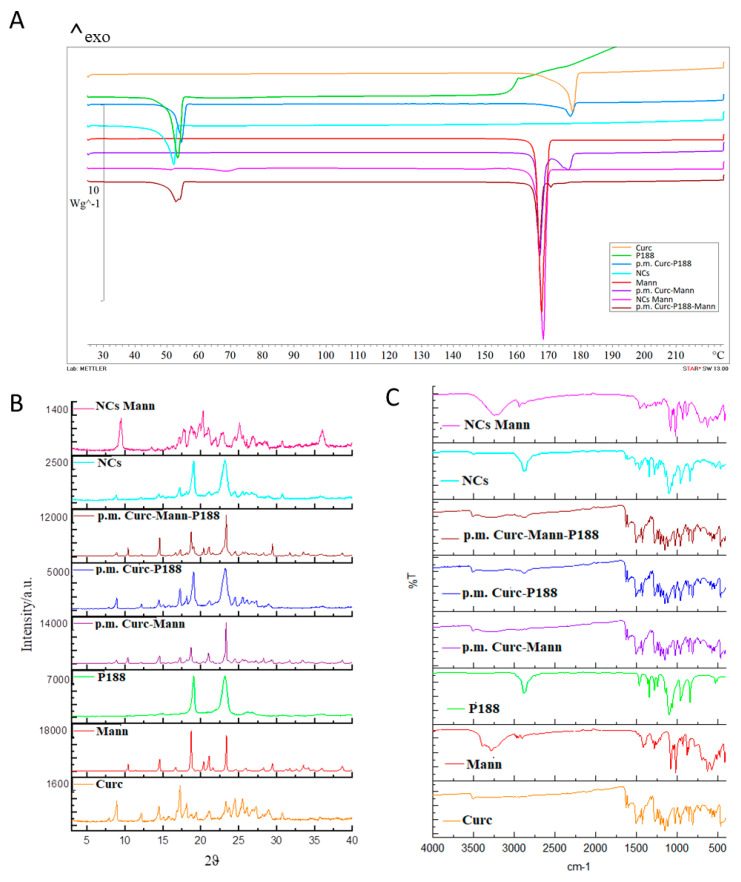
(**A**) Differential scanning calorimetric (DSC) thermograms of curcumin NCs with and without mannitol, curcumin (Curc), Poloxamer 188 (P188), mannitol (Mann) and the physical mixture (p.m.) of curcumin-mannitol and curcumin-poloxamer 188; and curcumin-poloxamer 188-mannitol. (**B**) XRPD spectra of the samples. Each sample was reported with the specific max intensity; (**C**) FT-IR analysis of all samples tested. Details of named samples: curcumin (Curc); mannitol (Mann); poloxamer 188 (P188); physical mix curcumin and poloxamer 188 (p.m. Curc-P188); physical mix curcumin, mannitol and poloxamer 188 (p.m. Curc-Mann-P188); physical mix curcumin and mannitol (p.m. Curc-Mann); nanocrystals (NCs); nanocrystals with mannitol (NCs Mann).

The DSC thermograms of raw materials, physical mixture (p.m.) and optimized freeze-dried NCs with and without mannitol are reported in Figure 4A. The thermogram of curcumin was typical of a crystalline substance with a sharp endothermic peak at the onset temperature of 174.87 °C. A DSC thermograph of poloxamer 188 exhibited a sharp endotherm with an onset melting point at 52.12 °C (Figure 4A). The thermal curve of mannitol exhibited a sharp endotherm with an onset melting point at 164.88 °C.

Both mannitol–curcumin and poloxamer–curcumin kept unchanged their onset melting points in the physical mixture. The absence of the curcumin melting endotherm in both NC formulations with or without mannitol revealed drug amorphization during the freeze-drying process [70]. Amorphization is also proved by the appearance of glass transition at about 50 °C on the DSC curve of NCs Mann. The peak at 70 °C could be the molecular relaxation phenomenon that usually appears as a weak endothermic transition near the end of a glass transition; however, it is not a melting peak, but an enthalpy relaxation peak. Stresses built into the material as a result of processing are released when the material is heated through its glass transition. The reason this occurs at Tg is that the molecule goes from a rigid to a flexible structure and thus can move to relieve the stress.

P188 is not perfectly crystalline. Thus, amorphous regions in a semi-crystalline polymer will display Tg, while ordered domains will display a melting point.

XRPD spectra of the different samples are reported in Figure 4B. Pure compounds (curcumin, mannitol, and poloxamer) exhibited the typical diffractogram of crystalline substances, and peaks distances are characteristic of each substance. The diffractograms of the physical mixtures are derived from the additivity of the peaks of pure compounds. NCs are characterized by the presence of peaks of curcumin and poloxamer. The relative intensity is correlated to the ratio of the two compounds in the mix, and a certain decrease in the crystallinity degree could be assumed from the decrease in the curcumin relative intensity peaks. For the NCs with mannitol, the diffractogram is characterized by the presence of peaks of curcumin, poloxamer and mannitol. Even if a certain tendency to a crystallinity decrease can be assumed, the powder is still crystalline and no complete amorphization occurred during processing.

The results of the FT-IR analysis are reported in Figure 4C and the most significant peaks are indicated and attributed in Table 5. From this technique, it is possible to distinguish between pure compounds and mixtures. By attributing the peaks to the different functional groups, it is possible to identify the presence of the different compounds in the mixtures. No significant differences arose from physical mixtures and NCs. In particular, concerning the –OH stretching, significant peaks were could be attributed for the different compounds, and the only significant difference could be observed in NCs prepared in the presence of mannitol, where a broad band appear from nearly 3500–3000 cm^−1^. This broad band might depend on a possible intermolecular interaction between curcumin and mannitol such as a hydrogen bond interaction.

### 3.5. Morphological Evaluation of Optimized Freeze-Dried NCs

Morphological analysis was performed in order to investigate the effect of crystallization techniques on the morphology of curcumin.

**Figure 5:**
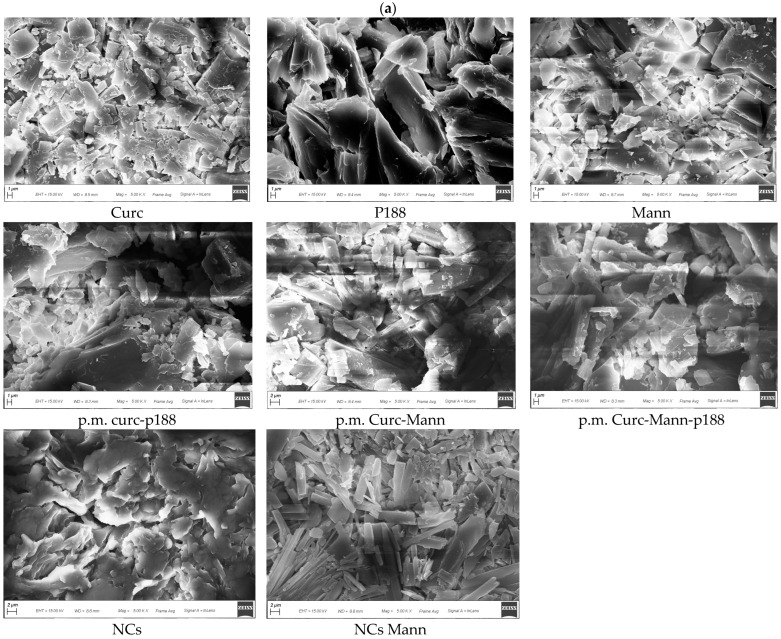

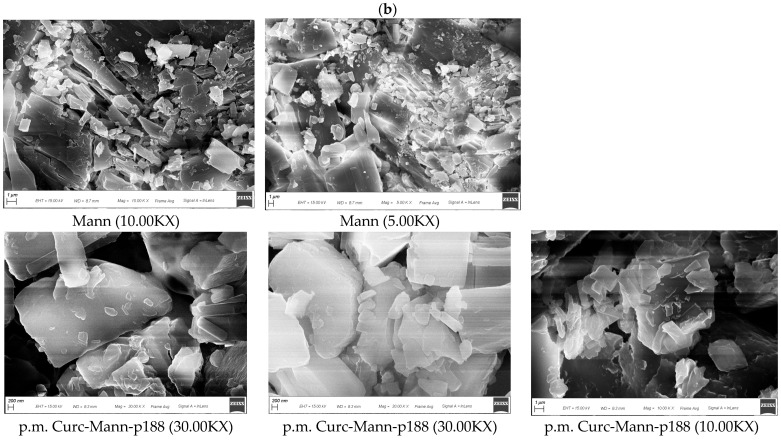
(**a**). Scanning Electron Microscopy of different samples (X5000). Details of named samples: curcumin (Curc); mannitol (Mann); poloxamer 188 (P188); physical mix curcumin and poloxamer 188 (p.m. Curc-P188); physical mix curcumin, mannitol and poloxamer 188 (p.m. Curc-Mann-P188); physical mix curcumin and mannitol (p.m. Curc-Mann); nanocrystals (NCs); nanocrystals with mannitol (NCs Mann); (**b**). Pictures showed images related to more magnification and different sample areas for Mann and p.m. Curc-Mann-P188.

Scanning electron photomicrographs are reported in Figure 5. Pure crystals of curcumin, mannitol and poloxamer appear as irregular particles, characterized by the presence of sharp edges. A similar morphology is found in physical mixtures. The morphology of NCs is totally different, characterized by agglomerated particles with round edges. In the presence of mannitol, the NC morphology was different and an elongated parallelepiped appeared; therefore, the mean dimensions registered by PCS did not refer to spherical particles.

During the sonication phase, spherical temporary particles could be broken down by the cavitation mechanism and this process could change their morphology. It has been demonstrated that curcumin could crystallize in three polymorphic forms, Form 1 (monoclinic structure) whereas Forms 2 and 3 exist as orthorhombic structures [65]. As reported in the literature, crystallization by the sonoprecipitation technique created orthorhombic forms (Form 2 or Form 3) with different crystal habits (depending on each additive used), while the crystallization of curcumin without any additive or ultrasound produced monoclinic forms with star-like crystals [65]. In the study of Yadav and Kumar (2014), freshly crystallized curcumin with and without any additive showed a spherical morphology, which was then distorted and converted into a rod-like crystal and then to slender prisms after 1.5 h; these crystals aggregate together to form flattened structures and platy-shaped crystals and after 3 h [71]. Our results partially corroborate the studies cited, because we found a different behaviour between curcumin NCs without and with cryoprotectant, even when prepared by using the same procedure. As stated, no cryoprotected curcumin NCs appeared as particles with a rounded shape; instead, curcumin NCs with mannitol seemed to adopt an orthorhombic form with flattened structures. Our findings confirm that curcumin can crystallize into different polymorphic structures. Both NCs were produced by using the sonoprecipitation method; thus, in our case, the presence of the additive mainly influences the curcumin crystallization state.

### 3.6. Storage Stability Of Curcumin Nanocrystals

The long-term stability studies were performed with the aim of assessing the storage stability of NCs at room temperature up to 6 months, with respect to particle size, ZP, PDI, drug content and thermotropic evaluation. The analyses were carried out after redispersing each sample (3.42 mg/mL curc in the final formulation) in demineralized water.

**Figure 6:**
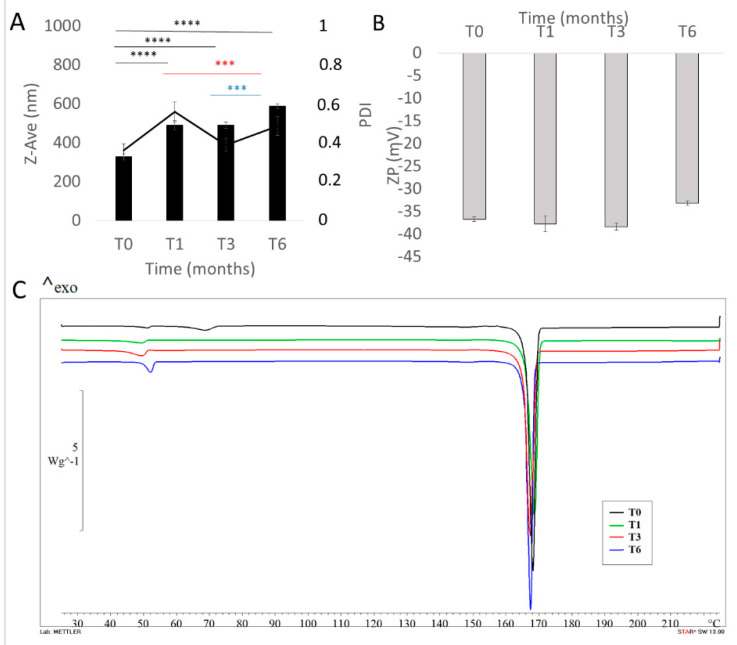
Stability study of lyophilized NCs stored at room temperature and analyzed by photon correlation spectroscopy (PCS): A) mean size and PDI: B) zeta potential (ZP) and C) DSC analysis up to 6 months. Tukey’s test for NC size and ZP. The black * symbol indicates statistical significance difference vs. T0 group; The red * symbol indicates statistical significance difference vs. T1 group; The blue * symbol indicates statistical significance difference vs. T3 group. Significance was defined as *** *p* < 0.001 **** *p*< 0.0001.

As shown in Figure 6A,B the Z-Ave of curcumin NCs were uniform, we registered a slight increase in particle size after the first month of storage, and no difference from 1 month of storage to 3 months. We observed a further increase in NC size after 6 months of storage, as highlighted by statistical analysis. We hypothesize that the increase in particle dimensions over time could be due to moisture accumulation during the storage of the samples or due to the crystallization of mannitol, which, during storage, could release sorbed water and promote product instability.

PDI values followed the same trend. No significant difference was registered for ZP values, suggesting that NCs maintained almost unchanged in their physico-chemical properties. However, the stability of freeze-dried curcumin NCs was also investigated by means of DSC (Figure 6C).

As reported previously, the appearance of glass transition at about 50 °C on the DSC curve of NCs Mann (T0) further proved the amorphization process. This phenomenon was not evident in the other thermograms registered (T1-T3-T6), in which we found a partial re-crystallization of poloxamer 188.

The partial re-crystallization of poloxamer 188 observed by DSC scans could suggest the formation of a shell/layer, protecting the drug. The basic principle that favors the nasal absorption of curcumin formulated as nanocrystals by using this surfactant is due to P188′s nature as a semi-crystalline triblock copolymer of poly(ethylene oxide) (PEO) and poly(propylene oxide) (PPO). In fact, PEO-based polymers have found uses in mucoadhesive drug delivery [72]. Poloxamers have a phase transition from liquid to mucoadhesive gel at body temperature and will therefore allow in-situ gelation at the site of interest. These surfactants are able to form liquid crystal phases when in contact with the mucus layer in order to avoid their rapid elimination by mucociliary clearance.

Na et al. (2010) studied the influence of different absorption enhancers on the intranasal absorption of isosorbide dinitrate (ISDN). It was found that P188 had better permeation-enhancing effect than that of hydroxypropyl-β-cyclodextrin and chitosans (at the same concentration). Both in situ and in vivo studies demonstrated that P188 played a key role in promoting the intranasal absorption of ISDN. Moreover, the mucoadhesion of poloxamer proceeds via an immediate adhesion of the polyoxyethylene chains to mucus through secondary forces, followed by their diffusion into mucus, where they undergo molecular interactions. In nasal ciliotoxicity test, all the absorption enhancers investigated showed good safety profiles. Taking both the enhancing effect and safety into account, the authors suggested P188 as the most promising intranasal absorption enhancer [45].

We found no critical difference in the thermograms of all sets of samples, suggesting no degradation or instability phenomena. This result was confirmed by a drug content assay, revealing a constant % amount of the drug (100% ± 0.08) in the lyophilized samples, showing the absence of curcumin chemical instability during the time intervals studied.

Thus, results obtained after 6 months showed that the optimized NC formulation was stable with respect to drug content, re-dispersibility, visual inspection, particle size and other parameters.

### 3.7. In Vitro Cell Uptake Study

Curcumin belongs to class IV of the BCS system; therefore, it is poorly soluble in water (~11 ng/mL) and poorly permeable. Therefore, to take advantage of the intranasal route as direct way to reach the brain, it is important to increase drug permeability, especially with OECs. The selection of this cell culture was performed on the basis of our scope, which is the intranasal administration of curcumin. OECs are glial cells of the olfactory system. They are able to secrete several neurotrophic growth factors, stimulate axonal growth and support the remyelination of damaged axons and they are also able to promote regeneration in the injured CNS [73]. We hypothesized that these cells exert a significant phagocytic activity and may exchange their contents with olfactory neuronal cells. This hypothesis is supported by our previous studies [64,73]. Moreover, in the study of Bonfanti et al. (2017), the authors investigated the protective effect of curcumin in OECs exposed to hypoxia and they found that curcumin stimulates cell viability in OECs grown in normal and hypoxic conditions [74]. Thus, our goal was to verify the possible uptake of the curcumin NCs and free curcumin into OECs to evaluate drug permeation and to consider NCs as a candidate formulation for drug nose-to-brain delivery. Intranasal administration of NCs could exerts a potential double effect acting at two different levels: the first action could be exercised locally on OECs, where, according to Bonfanti et al. (2017), curcumin NCs could protect these cells and stimulate their growth [74]; the second effect could be the direct transport of curcumin to the brain to exercise a central action [75].

**Figure 7:**
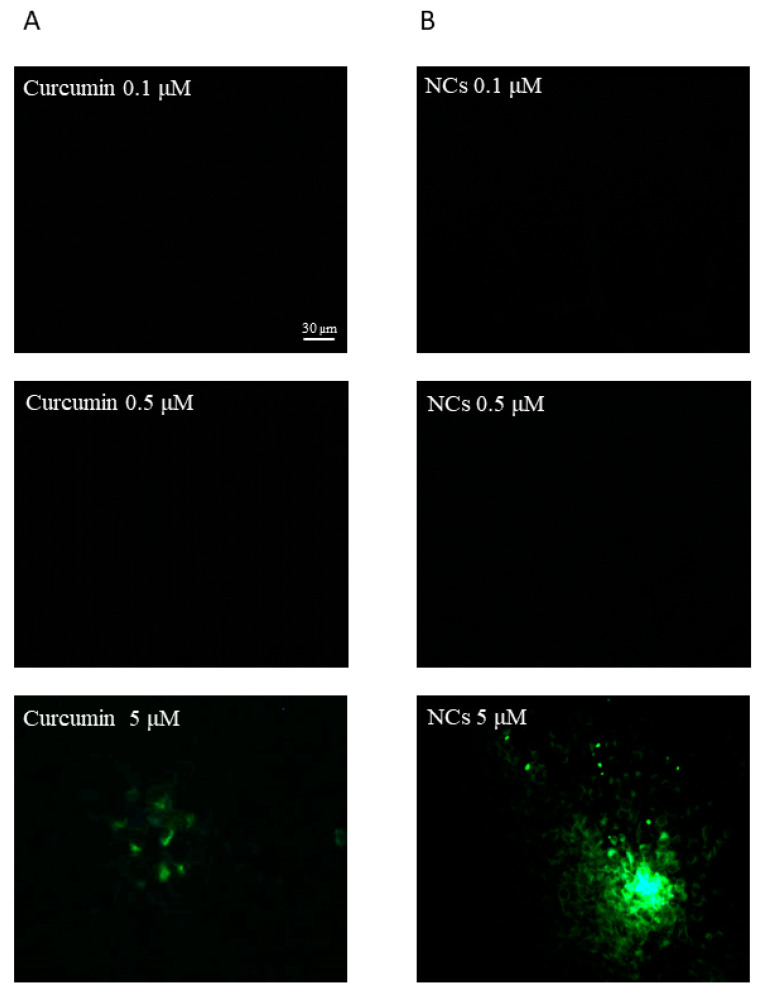
The internalization and uptake of curcumin (**A**): 0.1 µM; 0.5 µM; 5 µM; and Curcumin NCs (**B**): 0.1 µM; 0.5 µM; 5 µM into OECs. Scale bar: 30 µm.

The intrinsic green fluorescence of curcumin is a widely exploited factor in cellular uptake studies of curcumin. Curcumin has the advantage of being traced inside cells without being tagged by other fluorescent dyes. We confirmed the cellular internalization of curcumin formulated as nanocrystals by fluorescence microscopy in OECs. On the basis of Bonfanti et al. (2017), we selected specific concentrations (0.1–0.5–5 µM) of free drug and NCs to perform the cellular uptake evaluation. Free curcumin and curcumin NCs at the lowest concentrations did not exhibit any fluorescence (Figure 7A,B).

Curcumin NCs (5 µM) showed a high-intensity intracellular green fluorescence, proving that the OECs efficiently took up the NCs compared to the free curcumin at the same concentration (Figure 7B). However, the entry of free curcumin was not prominent in the cells (Figure 7A).

The satisfactory data obtained confirm that NCs can enhance drug permeability. We hypothesize that different factors affect the increased permeability of NCs compared to free curcumin on OEC internalization. A factor that could influence penetration is the surfactant added to the formulation of the NCs. In particular, in our case, the use of poloxamer 188 has proved to be advantageous for its double action as a stabilizer and penetration enhancer, as discussed in the work of Ying Li et al. (2016), where the same surfactant increased the permeation of the active substance in the rat nasal mucosa [46]. In fact, it worked both in maintaining NCs stability against aggregation phenomena and enhancing drug permeability.

These results corroborate Raveendran et al. (2013), in which similar observations on the effect of curcumin-encapsulated pluronic/polycaprolactone micelles in Caco cells were found [76].

In the study of Sahay et al. (2012), the authors studied the endocytosis mechanism of pluronic block copolymers with plasma membranes; they found that pluronic copolymers may interfere with membrane trafficking processes by inserting their hydrophobic PPO chains into lipid bilayers and decreasing membrane microviscosity [77]. Thus, the increased permeation of curcumin NCs in the OECs could be attributed to these mechanisms induced by the pluronic moiety.

Moreover, particle size is one of the most important parameters that plays a crucial role in determining cellular uptake efficiency and biodistribution. In drug formulation, the particle size of APIs is the primary variable for controlling the bioavailability of a drug.

The difference in the cellular uptake efficiency may be due to the variation in the physicochemical properties between free curcumin and curcumin NCs in terms of size, shape and surface hydropilicity. It has been reported in the earlier studies that cellular uptake decreases with increasing particle size [78,79]. Small particles (˂100 nm) can achieve the highest cellular uptake in certain cells, but can also induce tissue toxicity due to their high relative surface area and the capability to induce oxidative stress by disrupting cell organelles (such as mitochondria) [51]. Conversely, large particles (˃500 nm) will be taken up via phagocytosis, inducing humoral responses [80]. Finally, particles in the range of 100–300 nm can be internalized by cells through pinocytosis and we suppose this mechanism for our NCs.

According to our results, due to the large dimensions, free curcumin (~4500 µm), cannot permeate efficiently inside the cells; in fact, phagocytosis is the main uptake of particles larger than 500 nm. In addition to size, the shape of the particles also plays a pivotal role in the uptake pathway and trafficking. As discussed previously, curcumin NCs present plate- and needle-like shapes, this might be due to the layer of mannitol and poloxamer 188 on the NC surface.

Many studies reported the increased cellular uptake of non-spherical NPs compared to their counterparts, as summarised in the study of Zhao (2018) [81]. The results of a recent simulation also indicate that short nanorods with flat tips enter cells via a rocket mode with the long axis perpendicular to the plasma membrane. Therefore, we can suppose a similar tendency for our NC internalization mechanism.

These results have opened up exciting possibilities for the employment of shape-based approaches, to aid in avoiding the premature loss of nanocarriers via in vivo phagocytosis [82].

## 4. Conclusions

In conclusion, Box Behnken Design was a useful and reliable approach (error % 0.28) to design and optimize a new formulation based on curcumin nanocrystals with suitable properties for potential intranasal administration.

The optimized formulation was subjected to a chemical–physical and technological investigation, revealing that the nanocrystals present an average size of 328.7 nm ± 16.87 nm and a negative surface charge (−36.7 mV ± 0.45). The pH and osmolarity values confirm the compatibility of the nanosuspension in the nasal environment, as pH values between 5 and 6.5 and osmolarity within the range 85.47–341.88 mOsmol/L are well tolerated and do not damage the nasal cilia. Considering the chemical instability of curcumin in aqueous suspension, the final formulation was successfully converted into a powder through the freeze-drying process, the properties of the nanocrystals cryoprotected with mannitol 5% *w/v* remained almost unchanged after lyophilization, but underwent slight variation after 6 months in storage. After redispersion, curcumin NCs were highly concentrated in a small volume, which is suitable for IN instillation. DSC, IR and XRPD studies revealed the partial conversion of curcumin from a crystalline to an amorphous form. NCs appeared as elongated parallelepiped.

Uptake studies on OECs revealed a significant difference in the cellular uptake of nanocrystals (5 µM) and pure curcumin tested at the same concentration, as revealed by the great intensity in the fluorescence, showing that the NC formulation is able to increase the permeability of the drug in the olfactory glial cells. Therefore, curcumin NCs represent a potential formulation for nose-to-brain delivery application.

## Figures and Tables

**Table 1:** Factors and the corresponding levels investigated by the Box Behnken design.

Independent Variables	Type	Coded Factors	Levels	
			Low	High
Curc conc. (mg/mL)	Numeric	X_1_	1	3
Surfactant conc. (% *w/v*)	Numeric	X_2_	0.1	1
S/A ratio (*v/v*)	Numeric	X_3_	1:1	1:10
Surfactant type	Categoric	X_4_	PVP	
			Tween 80	
			Poloxamer 188	

**Table 2:** NC optimization.

Factors and Response	GOAL	Lower Limit	Upper Limit
Curc conc. (mg/mL)	maximize	1	3
Surfactant conc. (% *w/v*)	is in range	0.1	1
S/A ratio (*v/v*)	minimize	1	10
Surfacant type	is in range	Poloxamer 188	Tween 80
Size (nm)	minimize	216.1	572.8

**Table 3:** Lyophilization results of NCs with and without cryoprotectant.

Samples	Mean Size (nm) ± SD	Sf/Si	Appearance of the Ridispersed Suspension
NCs	809.2 ± 76.57	2.57	Clear, yellow
NCs MANN 5% w/V	328.7 ± 16.87	1.04	Very clear, intense yellow
NCs MANN 10% w/V	382.2 ± 15.62	1.21	Very clear, intense yellow
Curc	˂4500		Very weak color yellow

**Table 4:** Physico-chemical properties of optimized NCs.

Size (nm) ± SD	PDI ± SD	ZP (mV) ± SD	Drug Content (%) ± SD	pH ± SD	Osmolarity (mOsm/L) ± SD
328.7 ± 16.87	0.361 ± 0.03	-36.7 ± 0.45	100 ± 0.03	6.1 ± 0.02	145.1 ± 0.51

**Table 5:** Assignment of bands found in all sample spectra analyzed.

Assignment of Functional Group	Frequency (cm^−1^) in:							
	Curc	Mann	P188	p.m. Curc-Mann	p.m. Curc-P188	p.m. Curc-Mann-P188	NCs	NCs Mann
–OH stretching	3508.50	3390.70- 3282.35	-	3508.83 From Curc 3281.80 from Mann	3509.01 from Curc	3508.49 from Curc	3505.88	Broad band from 3500–3000
–CH_3_		2949.44	-	2971.78		3279.44 from Mann	2883.63	2936.99
–CH ALIPHATIC			2881.75		2884.48 from P188	2886.27 from P		

## Supplementary Materials

The following are available online at https://www.mdpi.com/1999-4923/12/5/476/s1, Figure S1: Correlation between predicted and actual values, Figure S2: Desirability of optimized formulation. Figure S3: Size distribution by intensity of redispersed NCs without cryoprotectant (A), with mannitol 5% *w/v* (B), and with mannitol 10% *w/v* (C) after freeze-drying process.
