# Correction: Bonaccorso et al. Optimization of Curcumin Nanocrystals as Promising Strategy for Nose-to-Brain Delivery Application. *Pharmaceutics* 2020, *12*, 476

**DOI:** 10.3390/pharmaceutics17101245

**Published:** 2025-09-24

**Authors:** Angela Bonaccorso, Maria Rosa Gigliobianco, Rosalia Pellitteri, Debora Santonocito, Claudia Carbone, Piera Di Martino, Giovanni Puglisi, Teresa Musumeci

**Affiliations:** 1 Department of Drug Sciences, University of Catania, V.le Andrea Doria, 6, 95125 Catania, Italy; debora.santonocito@outlook.it (D.S.); ccarbone@unict.it (C.C.); puglisig@unict.it (G.P.); tmusumec@unict.it (T.M.); 2 School of Pharmacy, University of Camerino, Via. S. Agostino 1, 62032 Camerino (MC), Italy; maria.gigliobianco@unicam.it (M.R.G.); piera.dimartino@unicam.it (P.D.M.); 3 Institute for Biomedical Research and Innovation, National Research Council, Via Paolo Gaifami 18, 95126 Catania, Italy; rosaliamariacristina.pellitteri@cnr.it

In the original publication [1], there was a mistake in Figure 5 as published. The mistake was related to the samples “mann” and “p.m. curc-mann-P188”. For the sample Mann, an image of low quality was erroneously inserted, and now, in Figure 5, all the acquisitions made, even those with better quality, are given for completeness. For the other sample (P.m. Curc-Mann-188), additional magnifications are provided for better clarity of the sample analyzed because for samples already inserted with the same magnification, there were effects on image quality due to hygroscopic sample conductivity. The corrected Figure 5 and legend appears below. The authors state that the scientific conclusions are unaffected. This correction was approved by the Academic Editor. The original publication has also been updated.

## Figures and Tables

**Figure 5:**
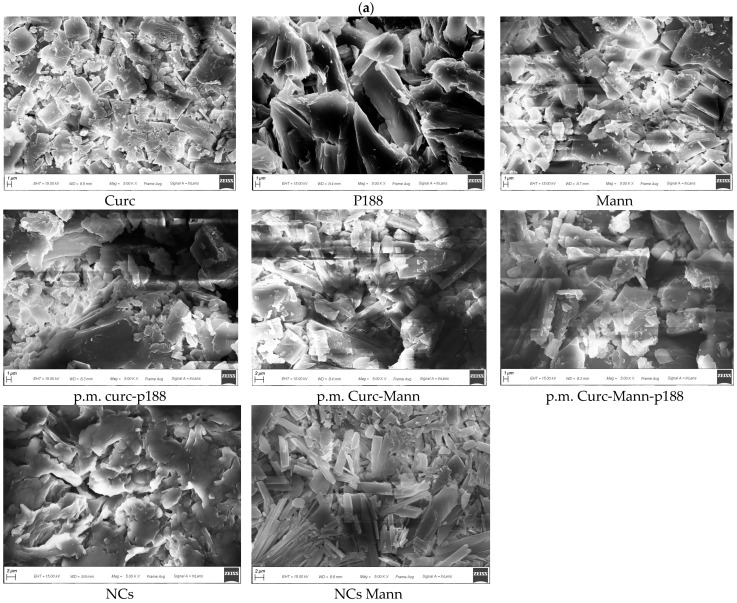

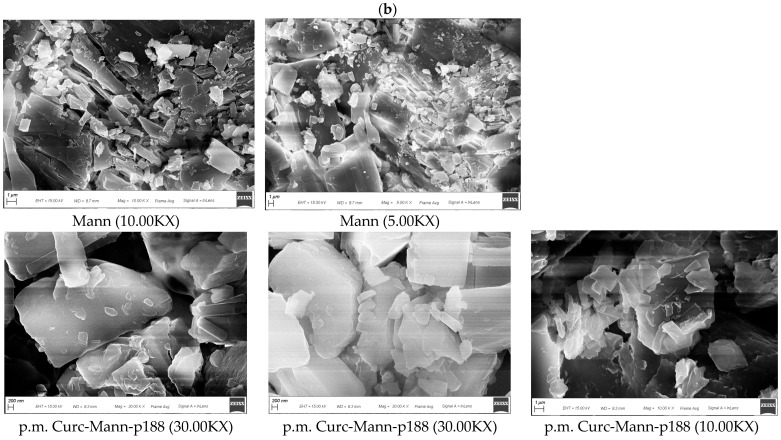
(**a**). Scanning Electron Microscopy of different samples (X5000). Details of named samples: curcumin (Curc); mannitol (Mann); poloxamer 188 (P188); physical mix curcumin and poloxamer 188 (p.m. Curc-P188); physical mix curcumin, mannitol and poloxamer 188 (p.m. Curc-Mann-P188); physical mix curcumin and mannitol (p.m. Curc-Mann); nanocrystals (NCs); nanocrystals with mannitol (NCs Mann); (**b**). Pictures showed images related to more magnification and different sample areas for Mann and p.m. Curc-Mann-P188.

## References

[1] Bonaccorso A., Gigliobianco M.R., Pellitteri R., Santonocito D., Carbone C., Di Martino P., Puglisi G., Musumeci T. (2020). Optimization of Curcumin Nanocrystals as Promising Strategy for Nose-to-Brain Delivery Application. Pharmaceutics.

